# Effect of a herbal extract powder (YY-312) from *Imperata cylindrica* Beauvois, *Citrus unshiu* Markovich, and *Evodia officinalis* Dode on body fat mass in overweight adults: a 12-week, randomized, double-blind, placebo-controlled, parallel-group clinical trial

**DOI:** 10.1186/s12906-017-1871-4

**Published:** 2017-07-28

**Authors:** Young-Gyu Cho, Ji-Hye Jung, Jae-Heon Kang, Jin Soo Kwon, Seung Pil Yu, Tae Gon Baik

**Affiliations:** 1 Department of Family Medicine, Seoul Paik Hospital, College of Medicine, Inje University, Mareunnaero 9, Jung-gu, Seoul, 04551 Korea; 2 Institute for Clinical Nutrition, Inje University, Seoul, Korea; 3 YuYu Pharma, Inc., Yuyu Building, 197 Dongho-ro, Jung-gu, Seoul, Korea

**Keywords:** *Imperata cylindrica* Beauvois, *Citrus unshiu* Markovich, *Evodia officinalis* Dode, Overweight, Weight loss, Anti-obesity agents

## Abstract

**Background:**

YY-312 is a herbal extract powder from *Imperata cylindrica* Beauvois, *Citrus unshiu* Markovich, and *Evodia officinalis* Dode, which have health promoting effects, including body fat reduction. We aimed to evaluate the efficacy and safety of YY-312 for body fat reduction in overweight adults.

**Methods:**

This was a 12-week, randomized, double-blind, placebo-controlled, parallel-group clinical trial performed in overweight Korean adults aged 19–60 years with a body mass index of 25.0–29.9 kg/m^2^. The daily dose of YY-312 was 2400 mg (containing 1800 mg of active herbal extract and 600 mg of cyclodextrin). Primary outcomes were reductions in body fat mass (BFM) and body fat percentage (BF%) after 12 weeks. Secondary outcomes included reductions in body weight and waist circumference (WC) after 12 weeks.

**Results:**

After 12 weeks, BFM (1.6 kg vs. 0.1 kg; *P* = 0.023) and BF% (1.5% vs. -0.2%; *P* = 0.018) decreased significantly more in the YY-312 group than in the placebo group, as did body weight (2.7 kg vs. 1.0 kg; *P* = 0.014) and WC (2.2 cm vs. 0.8 cm; *P* = 0.049). All safety parameters were within normal limits; no serious adverse events occurred in either group.

**Conclusions:**

In a 12-week clinical trial in overweight adults, YY-312 resulted in significantly greater reduction in body fat vs. placebo, while being safe and well tolerated.

**Trial registration:**

cris.nih.go.kr: (KCT0001225).

## Background

Obesity is a condition involving excessive accumulation of body fat that may impair health. The global prevalence of obesity has risen dramatically, increasing more than 2-fold since 1980. In 2014, over 1.9 billion adults worldwide were overweight, of which more than 600 million were estimated to be obese [1]. Obesity contributes to the development of hypertension, dyslipidemia, type 2 diabetes mellitus, coronary artery disease, and stroke, as well as overall mortality [2]. Obesity also leads to an increase in socioeconomic burden. The total socioeconomic costs of overweight and obesity in Korean adults in 2005 were estimated to be approximately US$1.8 billion, equivalent to 3.7% of the national health care expenditure for that year [3]. Hammond et al. [4] suggested that the total annual economic costs associated with obesity in the United States are in excess of US$ 215 billion. Development and implementation of cost-effective interventions for obesity prevention and management are essential to reduce the huge economic burden of obesity [5].

Treatment of obese patients requires a multifaceted approach, including dietary therapy, regular physical activity, behavioral therapy, and/or pharmacotherapy [6]. Comprehensive lifestyle intervention is foundational to obesity management, and adjunctive pharmacotherapy may be considered for individuals who are unable to achieve or maintain weight loss with comprehensive lifestyle intervention and have a body mass index (BMI) ≥30 kg/m^2^, or ≥27 kg/m^2^ with comorbidity [7]. Although the addition of weight loss medications to a lifestyle modification intervention can help obese individuals achieve greater weight loss, their body weight can rebound if they stop taking the medications. Since the withdrawal of sibutramine in 2010 because of the risk of serious cardiovascular adverse events, concerns about the safety of anti-obesity medications have led to a steady decline in prescription and use of these medications [8]. Due to the high costs, serious complications, and limited duration of effectiveness of anti-obesity drugs, there has been growing interest in and use of relatively inexpensive, safe, and effective functional food products from natural sources that are capable of aiding weight loss [9, 10].

Plants are considered good natural sources of bioactive compounds with potential anti-obesity properties [11, 12]. These plant-derived anti-obesity compounds induce weight loss through various mechanisms, including regulating lipid metabolism, suppressing food intake, and stimulating energy expenditure [10, 11]. However, there is still a paucity of data on the efficacy and safety of herbal plant preparations in obesity treatment. In order to provide obese patients with accurate and reliable information about effective and safe natural anti-obesity agents, there is a need for high-quality studies on the efficacy and safety of natural herbal products that claim to exert a weight reducing effect [13, 14]. In Korea, it is possible for a health functional food with body fat reducing effects to be approved for use after review, by the Ministry of Food and Drug Safety, of results of a clinical trial on the efficacy and safety of the product [15].

YY-312 is a herbal extract powder from *Imperata cylindrical* Beauvois, *Citrus unshiu* Markovich, *Evodia officinalis* Dode [16]. These plants have been commonly used as medicinal herbs in Korea, and have been reported to have health promoting effects, including reduction of body fat. Evodiamine, a major alkaloidal compound extracted from *Evodia officinalis* Dode, was thought to elicit anti-obesity effects through uncoupling protein-1 (UCP1) thermogenesis, but it was also suggested to have the potential to prevent obesity by inhibiting adipocyte differentiation through stimulating the extracellular signal regulated kinase (ERK)/mitogen activated protein kinase (MAPK) signaling pathway [17]. *Citrus unshiu* Markovich, the peel of immature citrus fruit in the Rutaceae family, is known to have plenty of flavonoids [18]. Citrus peel extracts have been reported to exert an anti-obesity effect through the promotion of β-oxidation and lipolysis in adipose tissue [19]. *Imperata cylindrical* Beauvois, the root of cogongrass in the Poaceae family, is known to have potent anti-oxidant activity due to its abundant polyphenols [20].

A previous study showed that YY-312 has an anti-obesity effect in high-fat diet (HFD)-induced obese mice and that it suppresses adipocyte differentiation in 3 T3-L1 cells [16]. However, it can be ascertained only through human clinical trials whether the individual ingredients in YY-312 have a synergistic effect in the human body, or whether their interactions augment toxicity. Hence, this randomized controlled trial was conducted to evaluate the efficacy and safety of YY-312 for body fat reduction in overweight Korean adults.

## Methods

### Study design

This study was designed as a 12-week, randomized, double-blind, placebo-controlled, parallel-group clinical trial to evaluate the body fat reducing effect and safety of YY-312. The study protocol was approved by the institutional review board of Seoul Paik Hospital, Inje University, before the trial began (IRB no. SIT-2013-335).

### Subjects

Korean adults aged 19 to 60 years, with a BMI of 25.0–29.9 kg/m^2^ were recruited between April and August 2014, by advertisements on the Seoul Metro. All study participants gave written informed consent to take part in the clinical trial. We excluded: individuals who were suspected of having a medical condition (identified by medical history, physical examination, and/or laboratory tests) that prevented them from participating in the trial, individuals who were suspected of having a major psychiatric disorder, individuals who were taking drugs or herbal preparations known to significantly affect body weight, pregnant or breastfeeding women, and/or individuals who had recently participated in another clinical trial. The full inclusion and exclusion criteria are displayed in Table 1.

**Table 1:** Inclusion and exclusion criteria

*Inclusion Criteria*
Age: 19–60 years
Body mass index: 25.0–29.9 kg/m^2^
Signed written informed consent
*Exclusion Criteria*
Blood pressure ≥ 160/100 mmHg or treated with diuretics or β-blockers
Fasting plasma glucose ≥126 mg/dL or treated with oral hypoglycemic agents or insulin
Fasting serum triglyceride level > 600 mg/dL
Serum AST or ALT level ≥ 3 times the upper limit of normal
Serum creatinine level ≥ 1.5 times the upper limit of normal
Thyroid-stimulating hormone ≤ 0.1 μU/mL or ≥ 10 μU/mL
Use of drugs, herbs or supplements known to significantly affect body weight (anti-obesity agents, anti-depressant drugs, glucocorticoids, laxatives, oral contraceptives, female hormones, etc.) in the past 3 months
Significant cardiac events within the past 6 months
History of malignancy within the past 5 years
Allergic disease, including bronchial asthma
Cardiac disease, cerebrovascular disease, pulmonary disease, endocrine disease, renal disease, gout, porphyria
Gastrointestinal disease, liver disease, gallbladder disease, pancreatic disease, malabsorption syndrome
Psychiatric disorder, including major depression, schizophrenia, bulimia, alcohol abuse, or substance abuse
History of abdominal adhesion after surgery
History of obesity surgery
Weight loss greater than 4 kg within the past 3 months
Pregnant or breastfeeding women
Women of childbearing age not adhering to acceptable forms of contraception
Participants in another clinical trial within the past 1 month

*AST* Aspartate aminotransferase, *ALT* Alanine aminotransferase

Sixty-seven individuals applied to participate in the trial and were assessed for eligibility. Of these, 7 did not meet study criteria and were excluded. The 60 remaining participants were randomized into the YY-312 group or the placebo group, with 30 participants in each group.

### Sample size and randomization with double blinding

Sample size was estimated by using two-sided two-sample t-test. Sample size of 30 for each group was obtained with a power of 68% to detect a difference in body fat percentage (BF%) of 1.7% and with a power of 64% to detect a difference in body fat mass (BFM) of 1.5 kg with an alpha of 0.05.

The study participants were randomly assigned to the YY-312 group or the placebo group in a 1:1 ratio according to a random allocation table compiled using a random number generator in a statistics program. Depending on their allocated group, the participants received either the active supplement or the placebo - with identical external appearance, mass, and taste. All participants and researchers were kept blinded to the group allocations until the end of the clinical trial.

### Study products

The study products were manufactured by the Central Research Institute, YuYu Pharma, Inc. (Suwon, Korea). YY-312 contains three kinds of powdered herbal extract, *Imperata cylindrica* Beauvois, *Citrus unshiu* Markovich, and *Evodia officinalis* Dode in a ratio of 5:2:3. The production process was standardized to maintain consistent amount of marker compounds, p-coumaric acid (0.247 mg/g), hesperidin (12.60 mg/g), and evodiamine (1.682 mg/g) in YY-312. The study products were pale yellow-colored, oblong-shaped, film-coated tablets. A YY-312 tablet includes 300 mg of active herbal extract and 100 mg of cyclodextrin for enhancement of solubility. A placebo tablet is indistinguishable with a YY-312 tablet. The participants were asked to take 3 tablets twice a day (after breakfast and supper). Thus, the daily dose of YY-312 was 2400 mg (containing 1800 mg of active herbal extract and 600 mg of cyclodextrin). The daily dose of YY-312 was calculated from an animal study with C57BL/6 mice [16] assuming body weight of the overweight to be 75 kg (1800 mg/kg = 300 mg/kg × 0.08 × 75 kg).

### Diet and physical activity counseling

All participants in both groups were instructed to reduce their energy intake by 500 kcal/day from their usual diet and to maintain their usual level of physical activity. At each visit, all participants were asked to submit a diary in which they recorded all food intake and physical activities for at least 3 days (including 1 weekend day). A trained dietitian reviewed all the diaries and provided counseling on diet and physical activity for the participants.

### Measurements

The participants visited the hospital at baseline, after 6 weeks, and after 12 weeks to undergo physical examination, anthropometric measurements, and laboratory tests. Anthropometric variables, including height, weight, and waist circumference (WC) were measured, and the BMI was calculated by dividing body weight (kg) by height (m) squared. Body composition including BFM, lean body mass (LBM), and BF% were assessed at baseline and after 12 weeks using dual-energy X-ray absorptiometry (Prodigy® DXA Lunar, GE Healthcare, Madison, WI, USA). All DXA measurements were performed by trained technicians after urinating and removing all metallic accessories. The participants were asked to fast for at least 8 h prior to DXA measurement.

Total cholesterol, triglycerides, high-density lipoprotein (HDL) cholesterol, low-density lipoprotein (LDL) cholesterol, and fasting plasma glucose (FPG) were measured to evaluate metabolic changes following changes in body composition.

In order to evaluate the safety of YY-312, the participants underwent tests for blood pressure and pulse rate measurement, electrocardiography, liver function tests (aspartate aminotransferase, alanine aminotransferse, γ-glutamyl transpeptidase, alkaline phosphatase, total bilirubin, total protein, albumin), renal function tests (blood urea nitrogen, creatinine), complete blood count test, electrolyte tests (sodium, potassium, chloride, calcium, phosphate), and urinalysis. Adverse reactions were monitored at each visit, and all unused study products were counted to assess adherence.

### Statistical analysis

The primary outcomes were reductions in BFM and BF% after 12 weeks, and the secondary outcomes included reductions in body weight and WC after 12 weeks. Independent sample t-tests were used to compare efficacy outcomes between the two groups, while the within-group changes were evaluated using paired sample t-tests. All statistical analyses were performed using IBM SPSS version 21.0 (SPSS Inc., Chicago, IL, USA), and results with a *P*-value <0.05 were considered to be statistically significant.

## Results

### Participants flow

**Fig. 1:**
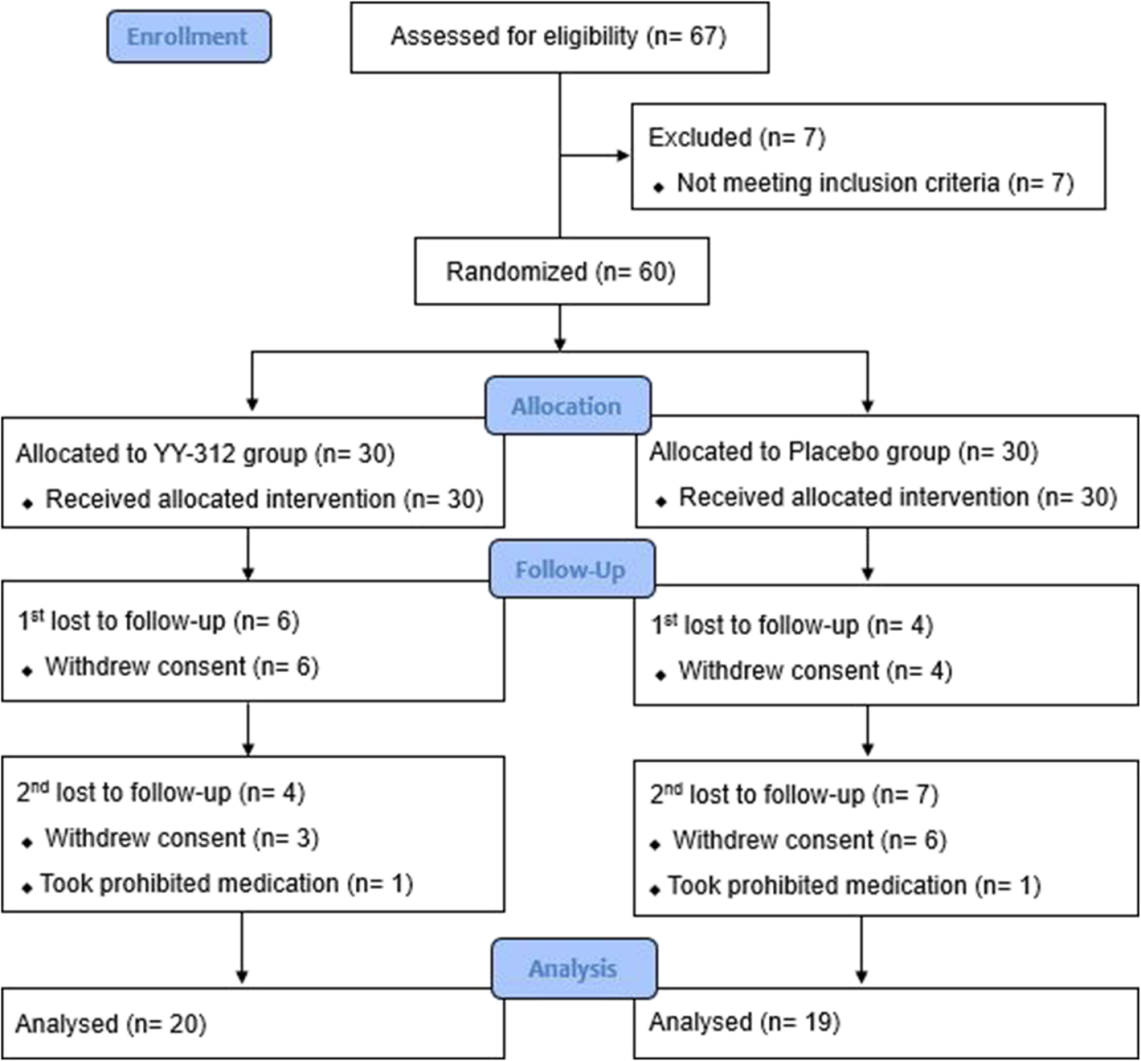
Flow diagram of study participants

Of the 67 individuals assessed for eligibility, 7 did not meet study criteria. The 60 remaining participants were randomly allocated to the YY-312 group or the placebo group with 30 participants in each. After 12 weeks, 21 participants (10 in the YY-312 group and 11 in the placebo group) dropped out prematurely due to withdrawal of consent (unvisited hospital or contact interruption) or the use of prohibited concomitant medication. Thus, 20 participants in the YY-312 group and 19 participants in the placebo group completed the 12-week clinical trial (Fig. 1).

### Baseline characteristics

The baseline characteristics of study participants are presented in Table 2. There were 10 men (33.3%) and 20 women (66.7%), with a mean age of 39.5 ± 11.2 years in the YY-312 groups, and 8 men (26.7%) and 22 women (73.3%), with a mean age of 41.7 ± 11.1 years in the placebo group. No significant differences were found between the 2 groups in the baseline measurement values for demographics, anthropometry, body composition, laboratory tests, and the daily energy intake. There were no differences in daily energy intake between two groups at 6 and 12 weeks (YY-312 group: 1314.7 kcal at 6 week, 1311.8 kcal at 12 week, placebo group: 1365.1 kcal at 6 week, 1152.1 kcal at 12 week).

**Table 2:** Baseline characteristics of study participants

	YY-312 group (*n* = 30)	Placebo group (*n* = 30)	*P*-value*
Sex			0.573
Male	10 (33.3%)	8 (26.7%)	
Female	20 (66.7%)	22 (73.3%)	
Age (years)	39.5 ± 11.2	41.7 ± 11.1	0.441
Height (cm)	165.9 ± 9.8	162.9 ± 9.9	0.234
Weight (kg)	75.0 ± 11.0	72.6 ± 10.8	0.400
BMI (kg/m^2^)	27.1 ± 1.5	27.2 ± 1.2	0.692
WC (cm)	88.3 ± 6.5	89.0 ± 7.1	0.705
Body fat (%)	32.2 ± 5.5	32.9 ± 4.6	0.562
BFM (kg)	22.6 ± 3.8	22.6 ± 3.6	0.961
LBM (kg)	48.6 ± 10.0	46.5 ± 8.8	0.392
T-Chol (mg/dL)	202.4 ± 40.2	206.8 ± 44.1	0.689
TG (mg/dL)	111.0 ± 58.6	126.4 ± 107.5	0.493
HDL-C (mg/dL)	53.1 ± 10.1	57.2 ± 10.5	0.128
LDL-C (mg/dL)	128.4 ± 31.5	126.8 ± 34.1	0.848
FPG (mg/dL)	91.5 ± 10.3	91.1 ± 7.7	0.843
AST (IU/L)	21.4 ± 5.5	22.2 ± 5.4	0.572
ALT (IU/L)	19.4 ± 6.9	22.1 ± 9.7	0.223
BUN (mg/dL)	11.8 ± 2.7	13.6 ± 4.1	0.051
Creatinine (mg/dL)	0.7 ± 0.2	0.7 ± 0.1	0.663
SBP (mmHg)	122.7 ± 12.4	119.9 ± 16.5	0.455
DBP (mmHg)	77.4 ± 9.5	75.5 ± 9.2	0.435
Energy intake (Kcal/day)	1511.6 ± 485.9	1464.8 ± 289.5	0.654

Values are expressed as mean ± standard deviation or number (%)

*BMI* Body mass index, *WC* Waist circumference, *BFM* Body fat mass, *LBM* Lean body mass, *T-Chol* Total cholesterol, *TG* Triglyceride, *HDL-C* High-density lipoprotein cholesterol, *LDL-C* Low-density lipoprotein cholesterol, *FPG* Fasting plasma glucose, *AST* Aspartate aminotransferase, *ALT* Alanine aminotransferase, *BUN* Blood urea nitrogen, *SBP* Systolic blood pressure, *DBP* Diastolic blood pressure

*P-value by Chi-square test or independent samples *t*-test

### Efficacy assessment

**Fig. 2:**
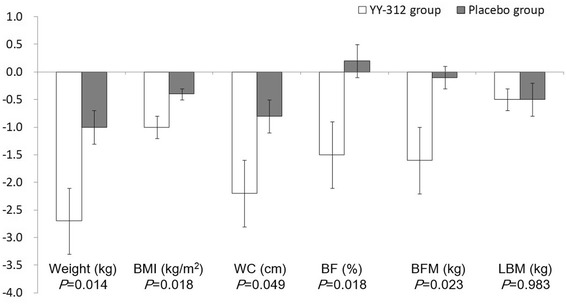
Mean changes in anthropometric and body composition variables after 12 weeks. Negative values correspond to decreases from baseline. Positive values correspond to increases from baseline. Error bars indicate SE. BMI: body mass index, WC: waist circumference, BF: body fat, BFM: body fat mass, LBM: lean body mass, SE: standard error

The anthropometric and body composition measures for each group at each visit are presented in Table 3. The primary efficacy endpoints were mean changes in BFM and BF% after 12 weeks. After 12 weeks, BFM in the YY-312 group decreased by 1.6 kg, whereas it reduced by only 0.1 kg in the placebo group (*P* = 0.023). BF% decreased by 1.5% in the YY-312 group, but increased by 0.2% in the placebo group (*P* = 0.018). The secondary efficacy endpoints were mean changes in body weight and WC after 12 weeks. While the YY-312 group showed a reduction in body weight of 2.7 kg after 12 weeks, the placebo group showed a decrease of 1.0 kg (*P* = 0.014). Furthermore, WC was reduced by 2.2 cm and 0.8 cm in the YY-312 and placebo groups, respectively, after 12 weeks (*P* = 0.049). Substantially greater decreases in BMI were observed in the YY-312 group than in the placebo group (1.0 kg/m^2^ vs. 0.4 kg/m^2^; *P* = 0.018). In contrast, the mean change in LBM was not significantly different between 2 groups (Fig. 2).

**Table 3:** Anthropometric and body composition measures by group and visit

	YY-312 group		Placebo group		*P*-value*
	n	Mean ± SD	n	Mean ± SD	
Weight (kg)					
Baseline	30	75.0 ± 11.0	30	72.6 ± 10.8	0.400
6 weeks	24	71.7 ± 10.5	26	72.2 ± 11.1	0.879
12 weeks	20	70.6 ± 11.4	19	69.9 ± 9.1	0.834
BMI (kg/m^2^)					
Baseline	30	27.1 ± 1.5	30	27.2 ± 1.2	0.692
6 weeks	24	26.2 ± 1.7	26	27.0 ± 1.3	0.097
12 weeks	20	25.8 ± 1.9	19	26.7 ± 1.4	0.135
WC (cm)					
Baseline	30	88.3 ± 6.5	30	89.0 ± 7.1	0.705
6 weeks	24	86.2 ± 5.8	26	88.5 ± 7.4	0.230
12 weeks	20	85.6 ± 6.7	19	87.4 ± 7.4	0.434
Body Fat (%)					
Baseline	30	32.2 ± 5.5	30	32.9 ± 4.6	0.562
12 weeks	20	30.0 ± 5.9	19	32.4 ± 5.5	0.199
BFM (kg)					
Baseline	30	22.6 ± 3.8	30	22.6 ± 3.6	0.961
12 weeks	20	20.1 ± 4.7	19	21.4 ± 3.5	0.335
LBM (kg)					
Baseline	30	48.6 ± 10.0	30	46.5 ± 8.8	0.392
12 weeks	20	47.4 ± 9.7	19	45.4 ± 8.6	0.494

*SD* Standard deviation, *BMI* Body mass index, *WC* Waist circumference, *BFM* Body fat mass, *LBM* Lean body mass

*P-value by independent samples *t*-test

Table 4 summarizes the FPG and serum lipid levels by group and visit. There were no significant differences between the 2 groups in the levels of FPG, total cholesterol, triglycerides, HDL cholesterol, or LDL cholesterol at any visit time-point.

**Table 4:** Fasting plasma glucose and serum lipid levels by group and visit

	YY-312 group		Placebo group		*P*-value*
	n	Mean ± SD	n	Mean ± SD	
FPG (mg/dL)					
Baseline	30	91.5 ± 10.3	30	91.1 ± 7.7	0.843
6 weeks	24	93.5 ± 13.3	26	90.2 ± 11.9	0.358
12 weeks	20	93.8 ± 13.0	19	94.3 ± 11.8	0.908
T-Chol (mg/dL)					
Baseline	30	202.4 ± 40.2	30	206.8 ± 44.4	0.689
6 weeks	24	204.4 ± 45.9	26	211.7 ± 46.5	0.579
12 weeks	20	203.5 ± 47.1	19	203.5 ± 33.8	0.999
TG (mg/dL)					
Baseline	30	111.0 ± 58.6	30	126.4 ± 107.5	0.493
6 weeks	24	111.3 ± 56.8	26	132.3 ± 80.6	0.295
12 weeks	20	107.4 ± 47.2	19	120.2 ± 67.9	0.496
HDL-C (mg/dL)					
Baseline	30	53.1 ± 10.1	30	57.2 ± 10.5	0.128
6 weeks	24	48.6 ± 8.7	26	56.0 ± 11.3	0.013
12 weeks	20	49.6 ± 8.3	19	55.3 ± 9.7	0.052
LDL-C (mg/dL)					
Baseline	30	128.4 ± 31.5	30	126.8 ± 34.1	0.848
6 weeks	24	125.9 ± 32.2	26	125.6 ± 34.2	0.978
12 weeks	20	128.0 ± 33.5	19	124.0 ± 28.2	0.690

*SD* Standard deviation, *FPG* Fasting plasma glucose, *T-Chol* Total cholesterol, *TG* Triglyceride, *HDL-C* High-density lipoprotein cholesterol, *LDL-C* Low-density lipoprotein cholesterol

*P-value by independent samples *t*-test

### Safety assessment

All safety parameters, including blood pressure, liver function tests, and renal function tests, were within normal ranges from baseline until the end of the study, with no significant differences between the 2 groups (Table 5). A total of 28 adverse events (12 in the YY-312 group and 16 in the placebo group) were reported over the study period, but there were no serious adverse events. The most common adverse events were gastrointestinal symptoms, such as dyspepsia, nausea, epigastric soreness, diarrhea, and constipation, which accounted for 12 adverse events (4 in the YY-312 group and 8 in the placebo group). In addition, there were 7 cases of upper respiratory tract infections (3 in the YY-312 group and 4 in the placebo group), 4 cases of neurologic symptoms, such as headache and dizziness (2 in the YY-312 group and 2 in the placebo group), 2 cases of skin rash (both in the YY-312 group), 2 cases of musculoskeletal pain (1 in the YY-312 group and 1 in the placebo group), and 1 case of fatigue (only in the placebo group).

**Table 5:** Safety outcome variables by group and visit

	YY-312 group		Placebo group		*P*-value*
	n	Mean ± SD	n	Mean ± SD	
SBP (mmHg)					
Baseline	30	122.7 ± 12.4	30	119.9 ± 16.5	0.455
6 weeks	24	121.2 ± 13.6	26	118.3 ± 14.5	0.470
12 weeks	20	119.2 ± 12.1	19	115.8 ± 15.5	0.454
DBP (mmHg)					
Baseline	30	77.4 ± 9.5	30	75.5 ± 9.2	0.435
6 weeks	24	75.3 ± 8.0	26	75.0 ± 8.8	0.903
12 weeks	20	73.1 ± 10.4	19	74.8 ± 9.1	0.583
AST (IU/L)					
Baseline	30	21.4 ± 5.5	30	22.2 ± 5.4	0.572
6 weeks	24	20.8 ± 8.0	26	21.1 ± 4.6	0.878
12 weeks	20	20.3 ± 4.8	19	22.7 ± 5.8	0.154
ALT (IU/L)					
Baseline	30	19.4 ± 6.9	30	22.1 ± 9.7	0.223
6 weeks	24	19.3 ± 9.6	26	19.7 ± 7.3	0.882
12 weeks	20	19.8 ± 10.2	19	22.9 ± 10.7	0.360
BUN (mg/dL)					
Baseline	30	11.8 ± 2.7	30	13.6 ± 4.1	0.052
6 weeks	24	10.9 ± 2.9	26	13.1 ± 4.5	0.049
12 weeks	20	11.3 ± 2.5	19	12.6 ± 4.2	0.217
Creatinine (mg/dL)					
Baseline	30	0.7 ± 0.2	30	0.7 ± 0.1	0.663
6 weeks	24	0.8 ± 0.1	26	0.8 ± 0.1	0.875
12 weeks	20	0.8 ± 0.2	19	0 8 ± 0.1	0.430

*SD* Standard deviation, *SBP* Systolic blood pressure, *DBP* Diastolic blood pressure, *AST* Aspartate aminotransferase, *ALT* Alanine aminotransferase, *BUN* Blood urea nitrogen

*P-value by independent samples *t*-test

## Discussion

This clinical trial aimed to evaluate the body fat reducing effect and safety of YY-312, a herbal extract powder from *Imperata cylindrica* Beauvois, *Citrus unshiu* Markovich, and *Evodia officinalis* Dode, in overweight adults. The participants who took YY-312 for 12 weeks had significantly greater reductions in BFM, BF%, body weight, and WC than the participants who took the placebo. This is consistent with results from a previous study investigating the anti-obesity effects of YY-312 in HFD-induced obese mice. Mice that had been given YY-312 with a high fat diet for 10 weeks showed a 10.7% reduction in body weight and a 25.0% reduction in abdominal adipose tissue weight compared to control mice that had been fed only a high fat diet [16].

Herbal plants can have anti-obesity effects through a variety of mechanisms, including decreased lipid absorption, suppressed energy intake, stimulated energy expenditure, inhibited adipocyte differentiation, and enhanced lipolysis [10, 21]. There is little evidence that a natural compound with a single mechanism of action results in successful treatment of obesity. Thus, combinations of multiple natural compounds with different mechanisms of action — that may have synergistic effects — could be an effective approach to address obesity [21, 22]. *Imperata cylindrica* Beauvois, *Citrus unshiu* Markovich, and *Evodia officinalis* Dode (the active ingredients of YY-312) have all traditionally been used in Korea as medicinal herbs. There have been several recent reports regarding the health promoting effects of these individual herbs — including reduction in body fat. Therefore, combined administration of these herbs might produce a synergistic action in terms of anti-obesity effects in the human body.

Evodiamine is an alkaloid that is present in high concentrations in *Evodia officinalis* Dode, and it is noted to possess a broad spectrum of biological activities, including anti-obesity, anti-inflammatory, anti-microbial, and anti-cancer activity [23]. Kobayashi et al. [24] supposed that evodiamine elicits capsaicin-like anti-obesity effects through enhancement of lipolysis, activation of brown adipose tissue, and heat dissipation. However, Wang et al. [17] showed that evodiamine has potential to prevent diet-induced obesity even in UCP1-knockout mice, suggesting that it activates a UCP1-independent mechanism to prevent the development of diet-induced obesity. They reported that evodiamine, unlike capsaicin, increased ERK/MAPK phosphorylation and reduced the expression of peroxisome proliferator-activated receptor (PPAR)γ and CCAAT/enhancer binding protein (C/EBP)β, and thereby inhibited adipocyte differentiation.

*Citrus unshiu* Markovich is rich in various flavonoids and carotenoids [18]. Hesperidin, a major flavonoid abundant in *Citrus unshiu* Markovich, has been reported to inhibit the lipase activity from porcine pancreas and to increase fecal lipid excretion from rats [25]. β-cryptoxanthin, a major carotenoid abundant in *Citrus unshiu* Markovich, has been reported to suppress 3 T3-L1 adipogenesis via the down-regulation of mRNA expression of PPARγ, through retinoic acid receptor activation [26]. It was also reported to suppress hypertrophy of abdominal adipocytes in an obese mouse model [27]. Kang et al. [19] showed that citrus peel extract increased β-oxidation by activating the phosphorylation of AMP-activated protein kinase (AMPK) and acetyl-CoA carboxylase in mature 3 T3-L1 adipocyte and enhanced lipolysis by stimulating the phosphorylation of cAMP-dependent protein kinase and hormone-sensitive lipase in mature 3 T3-L1 adipocyte.

*Imperata cylindrica* Beauvois is rich in coumarins, triterpenoids, saccharides, and organic acids, and is known to have hemostatic, diuretic, anti-inflammatory, anti-bacterial, and anti-tumor effects. *Imperata cylindrica* Beauvois has been reported to have potent anti-oxidant properties that may be attributed to its abundant polyphenols [20, 28]. Although there is a paucity of data on the potential anti-obesity effects of *Imperata cylindrica* Beauvois, polyphenols can contribute to a reduction in body fat by various mechanisms, including inhibited adipocyte differentiation, increased adipocyte apoptosis, and decreased fat absorption in the gut [29].

Kang et al. [16] reported that treatment with YY-312 suppressed adipocyte differentiation in 3 T3-L1 cells. They explained that this was because YY-312 suppressed expression of the key adipogenic transcription factors, including PPARɣ, C/EBPα, and fatty acid synthase, and increased the phosphorylation of AMPK in 3 T3-L1 cells. YY-312 is also expected to have anti-obesity activity through enhanced fatty acid oxidation by AMPK activation. In addition to inhibiting adipocyte differentiation and promoting lipolysis, *Imperata cylindrica* Beauvois, *Citrus unshiu* Markovich, and *Evodia officinalis* Dode - the active ingredients of YY-312 - are known to have other anti-obesity properties, such as stimulating energy expenditure and decreasing lipid absorption. Hence, further studies are required to elucidate whether YY-312, as an extract from these herbs, has these additional anti-obesity properties.

Although combined administration of multiple natural herbs with various anti-obesity effects may increase the body fat reducing effects, the interactions between the herbs may cause unexpected side effects [30]. Nevertheless, all safety parameters in this trial, including blood pressure, liver function tests, and renal function tests, were within normal ranges from baseline throughout the duration of the study. In addition, there were no serious adverse events over the study period. Thus, it is thought that YY-312 is a safe and well-tolerated anti-obesity agent.

This study has some limitations, including the small sample size of 60 participants and the short-term duration of 12 weeks. Because this study was performed only in overweight adults, we could not determine the possible influence of YY-312 in non-overweight adults. However, this study is of value in that this is the first randomized controlled trial to evaluate the efficacy and safety of the herbal extract YY-312 for body fat reduction in overweight Korean adults.

## Conclusion

In conclusion, in this 12-week clinical trial in overweight adults, YY-312 resulted in significantly greater reductions in body fat compared to placebo, while being safe and well tolerated. In future studies, the long-term efficacy and safety of YY-312 should be examined in a larger number of participants.

## Abbreviations

BFM: Body fat mass
BMI: Body mass index
DXA: Dual-energy X-ray absorptiometry
FPG: Fasting plasma glucose
HDL: High-density lipoprotein
LDL: Low-density lipoprotein
WC: Waist circumference
